# Delivery of adipose-derived growth factors from heparinized adipose acellular matrix accelerates wound healing

**DOI:** 10.3389/fbioe.2023.1270618

**Published:** 2023-10-03

**Authors:** Jiangjiang Ru, Qian Zhang, Shaowei Zhu, Junrong Cai, Yunfan He, Feng Lu

**Affiliations:** Department of Plastic Surgery, Nanfang Hospital, Southern Medical University, Guangzhou, Guangdong, China

**Keywords:** acellular dermal matrix, adipocyte, decellularized adipose tissue, growth factor, wound healing

## Abstract

Dermal white adipocytes are closely associated with skin homeostasis and wound healing. However, it has not been fully investigated whether adipose-derived products improve wound healing. Here, we obtained adipose acellular matrix (AAM) and adipose-derived growth factors (ADGFs) from human adipose tissue and fabricated an ADGF-loaded AAM via surface modification with heparin. The product, HEP-ADGF-AAM, contained an adipose-derived scaffold and released ADGFs in a controlled fashion. To test its efficacy in promoting wound healing, mice with full thickness wound received three different treatments: HEP-ADGF-AAM, AAM and ADM. Control mice received no further treatments. Among these treatments, HEP-ADGF-AAM best improved wound healing. It induced adipogenesis *in situ* after *in vivo* implantation and provided an adipogenic microenvironment for wounds by releasing ADGFs. HEP-ADGF-AAM not only induced adipocyte regeneration, but also enhanced fibroblast migration, promoted vessel formation, accelerated wound closure, and enhanced wound epithelialization. Moreover, there was a close interaction between HEP-ADGF-AAM and the wound bed, and collagen was turned over in HEP-ADGF-AAM. These results show that HEP-ADGF-AAM might substantially improve re-epithelialization, angiogenesis, and skin appendage regeneration, and is thus a promising therapeutic biomaterial for skin wound healing.

## 1 Introduction

Impaired wound healing is challenging to treat and places patients at increased risk of wound complications that negatively impact their quality of life and increase healthcare expenditure (Wang et al., 2018; Davis et al., 2018; Okur et al., 2020). Wound dressings that are functionally and structurally similar to normal skin tissue play important roles in the treatment of deep and full-thickness wounds (Landriscina et al., 2015; Obagi et al., 2019). Recent advances in tissue engineering approaches in the field of extracellular matrix (ECM) research have led to the generation of promising scaffold dressings for the treatment of impaired wound healing (Maquart and Monboisse, 2014). Various market products have been developed from the naturally occurring ECM scaffold and approved as skin substitutes for skin wounds and burns (Tavelli et al., 2020).

Adipose tissue is abundant, easily harvested and manipulated, and can be decellularized and converted into a biological scaffold termed acellular adipose matrix (AAM) (Brown et al., 2011). With its unique biological and mechanical properties, AAM has previously become a promising biomaterial scaffold for soft tissue reconstruction (Wu et al., 2012). Furthermore, it is also demonstrated that high efficiency of heparinized AAM loaded with growth factors will promote adipose regeneration and neovascularization with in the long-term (Zhang et al., 2016; Zhang et al., 2016). In recent years, AAM has gradually become a promising approach for tissue repair and regeneration. In the full-thickness skin wound model, AAM-hydrogel accelerated wound closure and increased neovascularization (Chen et al., 2020). During the treatment of bone defects, AAM is defined as an alternative bone graft material with preserved ECM components (Ahn et al., 2022). AAM can be converted into a powder, solution, foam, or sheet for convenient implantation to repair various injuries (Chun et al., 2019; Gentile et al., 2020).

In order to obtain adipose-derived growth factors (ADGFs), the decellularized adipose ECM was collected and undergo a series of centrifugation and dialysis, collecting the supernatant and lyophilize finally (Chun et al., 2019). The type and composition of ADGFs can vary depending on different extract conditions (Choi et al., 2012). Furthermore, numerous growth factors in ADGFs may assist angiogenesis, including vascular endothelial growth factor (VEGF), hepatocyte growth factor (HGF) and stromal cell-derived factor-1 (SDF-1) (Murohara et al., 2009). ADGFs can also stimulate both collagen synthesis and cell viability of dermal fibroblasts, which improved the wrinkling and accelerated wound healing in animal models (Kim et al., 2009).

*In vivo* study of nude mice with full-thickness wounds showed that AAM promotes wound healing and that AAM combined with adipose stem cells (ASCs) elicits the best effects (Xia et al., 2020). Similarly, delivery of ASCs using a hydrogel biological scaffold derived from human decellularized adipose matrix accelerates chronic wound healing and increases neovascularization (Chen et al., 2020). These studies revealed the therapeutic potential of AAM to promote wound healing. However, these strategies require the use of AAM in combination with viable cells, which limits their wider application.

Our previous study showed that a growth factor-rich liquid extract prepared from human subcutaneous adipose tissue contains a wide variety of proangiogenic and proadipogenic factors, which efficiently induce angiogenesis and adipogenesis *in vitro* and *in vivo* (He et al., 2019). Reconstitution of a bioactive AAM by coating it with native ADGFs is a potential strategy for regenerative purposes. Heparin reversibly binds to proteins including growth factors and soluble ECM proteins. Researches demonstrated that heparin-binding domain II in fibrin (the provisional ECM during tissue repair) plays an important role in promiscuous high-affinity GF-binding domain, which may be one of fibrin’s main physiological functions, assisting in tissue repair (Martino et al., 2013). Enhancement of the protein-binding ability of a biomaterial using heparin is important for drug delivery systems (Wissink et al., 2000a; Wissink et al., 2000b; Wissink et al., 2001; Pieper et al., 2002).

Acellular dermal matrices (ADMs) are commonly used acellular products, which can capitalize on the properties of native ECM in case of inadequately healing in traumatic or chronic wounds in clinical contexts (Kirsner et al., 2015). After the application of ADMs, recipient cells were tightly preserved in the matrix structure by growth factors and gradually incorporated into the matrix (Kirsner et al., 2015; Boháč et al., 2018). Furthermore, a variety of cells invade into the ADMs and matrix begin to remodel, numbers of collagen and elastin increase, and angiogenesis is initiated (Boháč et al., 2018; Lee et al., 2015; Mirzaei-Parsa et al., 2019; Ye et al., 2016). In recent years, various ADMs have been developed from human cadaver (HADM), bovine (BADM) and porcine (PADM) tissues, which would be used in a variety of different clinical contexts with regard to different applications (Petrie et al., 2022).

In the present study, we aimed to functionalize AAM with heparin in order to enhance its ability to bind ADGFs. The capacity of heparinized ADGF-bound AAM (HEP-ADGF-AAM) to release growth factors and its effects on cellular functions were assessed. The efficiencies with which HEP-ADGF-AAM and AAM repaired full-layer wounds in comparison with acellular dermal matrix (ADM) were assessed, and particular attention was paid to dermal adipogenesis and wound healing.

## 2 Materials and methods

### 2.1 Fat harvesting and AAM preparation

**FIGURE 1:**
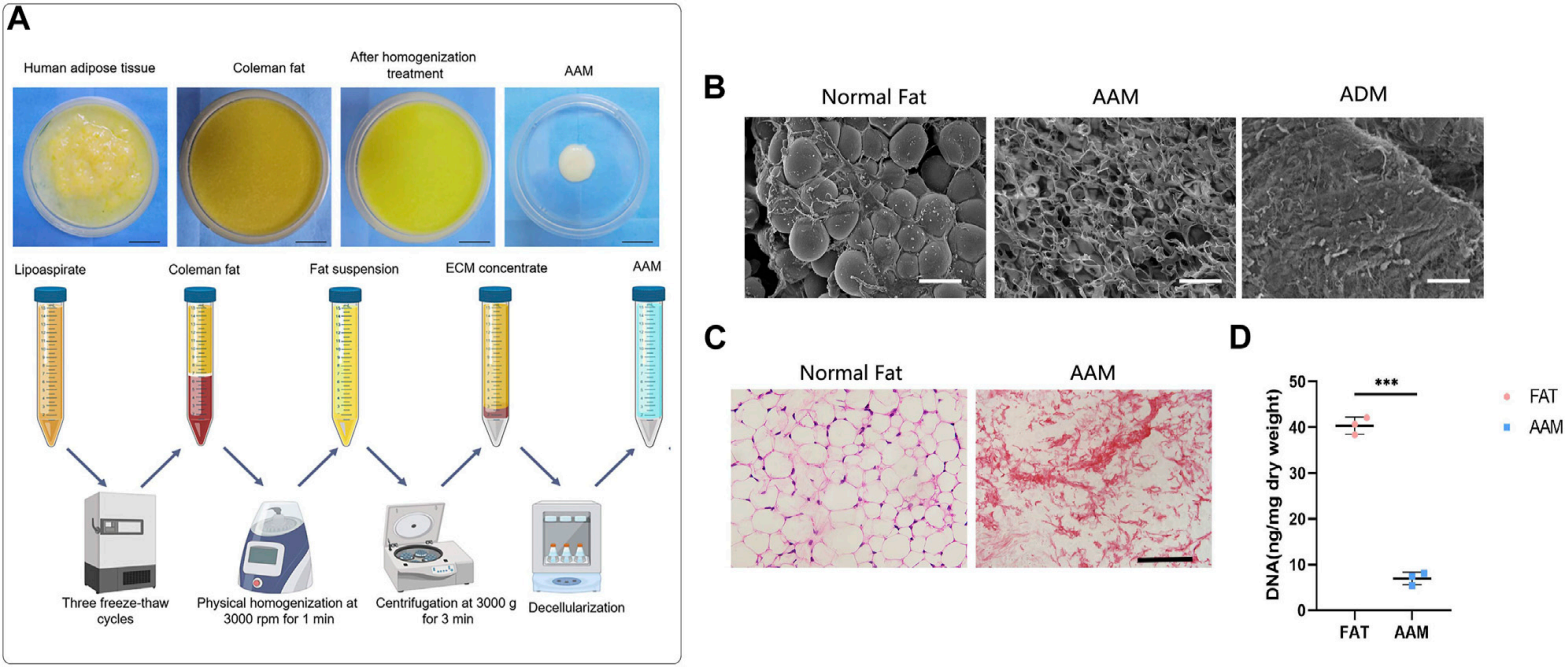
Preparation and characterization of AAM **(A)** Scheme of AAM preparation. **(B)** Scanning electron micrographs of AAM, ADM and normal fat tissue. Scale bar = 100 µm. **(C)** H&E staining of AAM and normal adipose tissue. Scale bar = 100 µm. **(D)** Quantification of the residual DNA content of AAM and normal adipose tissue. ***p < 0.001.

To prepare AAM, adipose tissue was obtained through liposuction in human patients at the department of Plastic and Cosmetic Surgery, Nanfang Hospital. The study protocol was approved by the Institutional Review Board of the Nanfang hospital. After obtaining informed consent, human adipose tissue was harvested from four healthy female patients (aged 22–34 years) undergoing abdominal liposuction. Fat was obtained by manual liposuction using a combination of intravenous general anesthesia and local anesthesia. Two-hole blunt-tipped liposuction needles with diameters of 2.5 and 3.0 mm and a lateral hole (2 mm diameter) were used for suction with a 10 mL screw-tipped syringe under low negative pressure. The detailed procedure of AAM preparation is shown in Figure 1A. Briefly, the tissue was subjected to three cycles (1 h each) of freezing and thawing (−80°C to 37°C) in balanced salt solution. Following centrifugation (2,000 × g, 3 min), the fat samples were homogenized using a specially made blender (Shanghai Tiangong Instruments Co., Ltd., Shanghai, People’s Republic of China) at 3,000 rpm for 1 min. After homogenization, the adipose suspension was collected and centrifuged again at 3,000 × g for 3 min to remove the free lipid content. After washing with PBS, the samples underwent 6 h of polar solvent extraction in 99.9% isopropanol to remove the lipid content. After three rinses with phosphate-buffered saline (PBS), the samples were decellularized with aqueous sodium deoxycholate (2%) for 12 h. After three rinses with PBS, the samples were disinfected with 4% ethanol containing 0.1% peracetic acid for 4 h and then rinsed three more times with PBS. The remaining ECM component (i.e., AAM) was soaked in PBS containing 1% penicillin and stored at −20°C.

### 2.2 Preparation of ADGFs and HEP-ADGF-AAM

Human ADGFs were prepared as previously described (He et al., 2019). The obtained adipose tissue was washed with PBS and centrifuged at 1,200 × g for 3 min to remove blood constituents. Then, the adipose tissue was mixed with an equal volume of PBS and physically emulsified by repeated transfer between two 10 mL syringes connected by a female-to-female Luer-Lock connector (1.4 mm internal diameter). Adipose tissue was transferred for 1 min at a constant rate (10 mL/s). After centrifugation at 1,200 × g for 2 min, the liquid portion from emulsified adipose tissue was passed through a 0.20 μm syringe filter (Xiboshi; DIONEX, USA) to remove cell and tissue debris, and was collected as ADGFs.

For loading of ADGFs, heparin and AAM were crosslinked as described previously (Wissink et al., 2001). Briefly, the carboxylic acid groups of heparin were activated with 1-ethyl-3 (3-dimethylaminopropyl) carbodiimide (EDC)/N-hydroxysuccinimide (NHS). One milligram of heparin (H-4784; Sigma, Steinheim, Germany) was activated with 1 mg EDC and 0.6 mg NHS in 500 mL of 0.05 M MES buffer (pH 5.6) for 10 min. The AAM specimen was immersed in this solution, which was then evacuated at 20 mmHg for ∼2 min to remove air from AAM. The reaction was allowed to proceed for 4 h at 37°C, after which AAM was extensively washed with 0.1 M Na_2_HPO_4_ (2 h), 4 M NaCl (four times in 24 h), and distilled water (five times in 24 h). After the modification procedure, AAM was frozen at −80°C overnight and lyophilized. Native and heparinized AAM was cut into discs (1 × 1 × 0.5 cm^3^) for experiments. The native and heparinized AAM samples were incubated with 5 mL ADGF solution for 90 min at room temperature and then washed with 5 mL PBS (two times for 5 min) to remove all non-bound ADGFs. Finally, HEP-ADGF-AAM and ADGF-AAM were frozen at −80°C overnight.

### 2.3 Evaluation of decellularized matrix

Both fat tissue and AAM samples were fixed in 4% paraformaldehyde and embedded in paraffin. Five-micrometer sections were then cut for hematoxylin and eosin (H&E) staining. In addition, residual DNA was extracted using a DNeasy kit (Qiagen, Valencia, CA, USA). DNA content (µg/mg wet weight) was then quantified with a microplate reader (Model 680; Bio-Rad, Hercules, CA, USA) at 260 nm and normalized to the initial wet weight of each sample.

### 2.4 Scanning electron microscopy (SEM)

Scanning electron microscopy (SEM) SEM was used to examine decellulized products and normal fats. Samples were fixed in 2% glutaraldehyde for 2 h, dehydrated using an ethanol series (30%–100%), critical point-dried, coated with gold, and examined under a scanning electron microscope (S-3000N; Hitachi Ltd., Tokyo, Japan). SEM images were captured using a digital camera (Canon Inc., Tokyo, Japan).

### 2.5 Collagen labeling of acellular products

To visualize the collagen turnover in the acellular products, the collagen components of ADM, AAM, and HEP-ADGF-AAM were labeled immediately after preparation according to a previously described protocol (Correa-Gallegos et al., 2019). Before being applied to wounds, ADM, AAM, and HEP-ADGF-AAM (500 mg) were incubated with 100 µM Alexa Fluor 647 NHS Ester (A20006; Thermo Fisher Scientific, Foster City, CA, USA) for 1 h at 25°C, and then washed three times with PBS.

### 2.6 Full-thickness skin wound model

The wound healing efficacy was evaluated using a full-thickness wound mouse model. Male nude mice aged 8–10 weeks and weighing 20–25 g were purchased from the Laboratory Animal Center of Southern Medical University. For wound healing experiments, mice were anesthetized and two circular full-thickness excisional skin wounds of 8-mm radius were made on the dorsum of mice. A prefabricated 12 mm silicone ring (inner ring radius of 8 mm) was placed around the wound and fixed using 4-0 silk sutures to prevent wound contraction (Lin et al., 2021). To compare *in vivo* wound healing efficacies, 48 nude mice were randomly divided into the HEP-ADGF-AAM, AAM, ADM (Beijing Jayyalife Biological Technology Co.,Ltd.), and control groups (*n* = 12 per group). Wounds were untreated in the control group, while covered with ADM, AAM, and HEP-ADGF-AAM dressings in other groups, respectively. All dressings had a diameter of 8 mm, and a thickness of 1 mm. Wounds are treated every 2 days and sterile dressings are changed daily. Photographs of the skin around the wound and healthy skin were taken at 5 and 14 days postoperatively (*n* = 6 per time point) and samples were taken for further histological analysis. At the end of the study, all mice were anesthetized by intraperitoneal injection of pentobarbital sodium (0.3%, 50 mg/kg). Cervical vertebra dislocation was used for euthanasia and death was confirmed by cessation of the heartbeat. Using the ImageJ software to quantify the wound area, wound healing is represented as follows: residual wound area/original wound area 100%. Researchers were blinded to the analysis of data from different groups.

### 2.7 Subcutaneous implantation of acellular products

To assess the adipogenic capacity of these acellular products *in vivo*, ADM, AAM, and HEP-ADGF-AAM (diameter of 8 mm and thickness of 3 mm) were subcutaneously inserted into the backs of nude mice, and then the skin was closed with 7-0 nylon sutures. Twelve weeks later, the implanted material was harvested and analyzed (*n* = 6 per group).

### 2.8 Histological assessment and immunohistochemical staining

Fresh samples of wound skin and subcutaneous tissue were fixed in formalin for histological assessment and immunohistochemical staining. Briefly, formalin-fixed samples were embedded and sliced into 5-μm sections. For histological assessment, tissue sections were deparaffinized in xylene, rehydrated through graded alcohol in phosphate-buffered saline (PBS), and then stained with a hematoxylin-eosin (HE) working solution. For immunohistochemical analysis, sections were dewaxed and rehydrated, then incubated in 3% H_2_O_2_ to block endogenous peroxidase. Immunohistochemical staining was performed using antibodies against vimentin (1:25, ab92547, Abcam, Cambridge, UK) and perilipin (1:500, GP29; Progen, Heidelberg, Germany). The vimentin + cells and perilipin + adipocytes were evaluated by counting the number of brown-labeled cells from six fields of each slide. For immunofluorescence staining, the sections were incubated with the following primary antibodies: guinea pig anti-mouse Perilipin (Progen, GP29, Germany) and rat anti-CD31 (1:200; Abcam, Cambridge, MA, USA), followed by the corresponding secondary antibodies. After DAPI staining (Sigma, D9542, USA), the sections were observed and photographed using a FV10i-W confocal laser scanning microscope (Olympus, Japan). Angiogenesis was evaluated by counting the number of fluorescent vessel-like structures from six fields of each slide.

### 2.9 *In vitro* growth factor release study

Upon immobilization of ADGFs onto native and heparinized AAM, the scaffolds were immersed in 2 mL PBS. Release of VEGF, HGF, bFGF, and EGF from HEP-ADGF-AAM and ADGF-AAM was assessed at 1, 3, 5 and 7 days. Supernatants were collected at designated time points and was analyzed using VEGF, HGF, bFGF, and EGF enzyme-linked immunosorbent assay (ELISA) kits (R&D Systems, Minneapolis, MN, USA) following the manufacturer’s instructions. The experiment was repeated three times.

### 2.10 Scratch-wound assay

Fibroblasts were cultured in six-well plates until reaching 100% confluence and a linear gap was generated using a sterile 200-μL pipette tip. Complete medium was then replaced with a serum-free medium supplemented with 2 mL of soak solution obtained by soaking ADM, AAM or HEP-ADGF-AAM within PBS for 24 h or serum-free medium (control group); the cells were then cultured for 0, 12 and 24 h. Three representative images of each scratched area were obtained using a light microscope (Nikon, Japan). Cell migration rate was analyzed using ImageJ software (NIH, MD, USA). All scratch-wound assays were carried out in triplicate.

### 2.11 Tube formation assay

The Matrigel (diluted 1:1 in PBS) was transferred to a 24-well plate and solidified by incubation at 37°C and 5% CO_2_ for 30 min. HUVECs (5 × 10^5^ per well) were then seeded on the Matrigel and treated with 2 mL medium (1:1, soak solution obtained by soaking ADM, AAM or HEP-ADGF-AAM within PBS for 24 h to endothelial cell basal medium). HUVECs treated with angiogenic factors [20 ng/mL vascular endothelial growth factor (VEGF) and 5 ng/mL basic fibroblast growth factor (bFGF)] served as a positive control, while cells treated with 2 mL medium (1:1, PBS to endothelial cell medium) were used as a negative control. Angiogenesis is evaluated by counting the number of tubular structures from six fields of each well. Tube formation assays were repeated three times.

### 2.12 Induction of adipogenic differentiation of ADSCs

ADSCs (1 × 10^5^ per well) were plated on 6-well plates and cultured overnight at 37°C and 5% CO_2_. The next day, cells in each well were rinsed and treated with 2 mL medium (1:1, supernatants from ADM, AAM or HEP-ADGF-AAM soak solution to human ADSC complete growth medium). ADSCs treated with 2 mL medium (1:1, PBS to human ADSC complete growth medium) were used as the negative control. The medium in all the groups was changed every 3 days. Adipogenic differentiation of ADSCs was examined for lipid accumulation by Oil Red O (ORO; Sigma-Aldrich, USA) staining. Cells were photographed using a Nikon E200 microscope and collected for gene expression analysis at days 21. Adipogenic induction assays were repeated three times.

### 2.13 qRT-PCR

Samples of wound beds and subcutaneous implants were quickly excised, immediately frozen in liquid nitrogen, and stored at −80°C. Total RNA was extracted from 50 mg frozen tissue using a RNeasy Lipid Tissue Mini Kit (Qiagen, Hilden, Germany), according to the manufacturer’s instructions. cDNA was amplified in 40 cycles using the QuantiTect Reverse Transcription Kit (Qiagen, Germany) and Rotor-Gene 3,000 Real-Time PCR Detection System (Corbett Research, Sydney, Australia). GAPDH was used as the reference gene. The primer sequences were as follows: peroxisome proliferator-activated receptor gamma (PPARG), forward 5′-TCG​CTG​ATG​CAC​TGC​CTA​TG-3′ and reverse 5′-GAG AGG​TCC​ACA​GAG​CTG​ATT-3′; CCAAT-enhancer-binding protein alpha (CEBPA), forward, 5′-CTT​GAT​GCA​ATC​CGG​ATC​AAA​C-3′ and reverse, 5′-CCC​GCA​GGA​ACA​TCT​TTA​AGT-3′; vascular endothelial growth factor A (VEGFA), forward 5′-TTA​CTG​CTG​TAC​CTC​CAC​C-3′ and reverse 5′-ACA​GGA​CGG​CTT​GAA​GAT​G-3′; basic fibroblast growth factor (bFGF), forward 5′- AGC​GGC​TGT​ACT​GCA​AAA​ACG​G-3′ and reverse 5′-CCT​TTG​ATA​GAC​ACA​ACT​CCT​CTC-3’. Expression levels were calculated using the 2^−ΔΔCT^ method.

### 2.14 Statistical analysis

Data were analysed using IBM SPSS (V22.0; IBM Corp., Armonk, NY, USA). An independent variable *t*-test was performed to compare the two mean values. For comparison of three or more means, one-way ANOVA was conducted, followed by the *post hoc* Tukey test. All results were expressed as mean standard deviation. The level of significance was set as *p* < 0.05.

## 3 Results

### 3.1 Physical characteristics of AAM

AAM was prepared as shown in Figure 1A. Scanning electronic microscopy (SEM) showed that AAM had a porous structure, whereas normal fat contained a large volume of connected adipocytes and ADM presented condensed collagen fibers (Figure 1B). Hematoxylin and eosin (H&E) staining showed that AAM only contained ECM and lacked any cellular components (Figure 1C). The residual DNA content of AAM (7.42 ± 2.25 ng/mg) was significantly lower than that of normal adipose tissue (40.65 ± 3.12 ng/mg) (*p* < 0.001) (Figure 1D), indicating that cellular components were effectively removed from AAM.

### 3.2 Controlled release of ADGFs from HEP-ADGF-AAM

**FIGURE 2:**
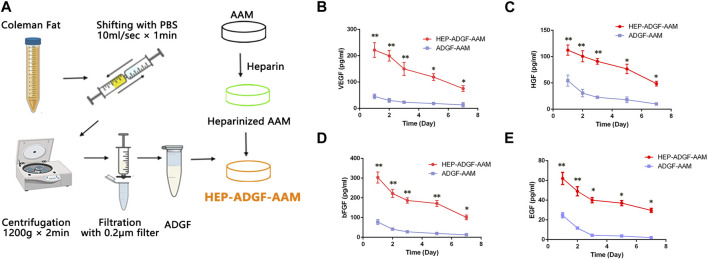
Preparation and characterization of ADGFs and HEP-ADGF-AAM **(A)** Scheme of ADGFs and HEP-ADGF-AAM preparation. ELISAs of **(B)** VEGF, **(C)** HGF, **(D)** bFGF, and **(E)** EGF in soak solution obtained by soaking ADGF-AAM or HEP-ADGF-AAM within PBS at 1, 3, 5 and 7 days. *p < 0.05, **p < 0.01.

HEP-ADGF-AAM was prepared as shown in Figure 2A. To assess the efficiency with which HEP-ADGF-AAM released ADGFs, growth factors released from HEP-ADGF-AAM were quantified using ELISA and compared with those released by ADGF-bound AAM (ADGF-AAM). ELISA of VEGF (Figure 2B), hepatocyte growth factor (HGF) (Figure 2C), bFGF (Figure 2D), and endothelial growth factor (EGF) (Figure 2E) showed that release of these growth factors was sustained after a peak in the HEP-ADGF-AAM group, with relatively high concentrations maintained for up to 7 days, while the concentrations of these growth factors remained low after Day 2 in the ADGF-AAM group.

### 3.3 Comparison of the *in vivo* wound healing efficacies of different acellular wound dressings

**FIGURE 3:**
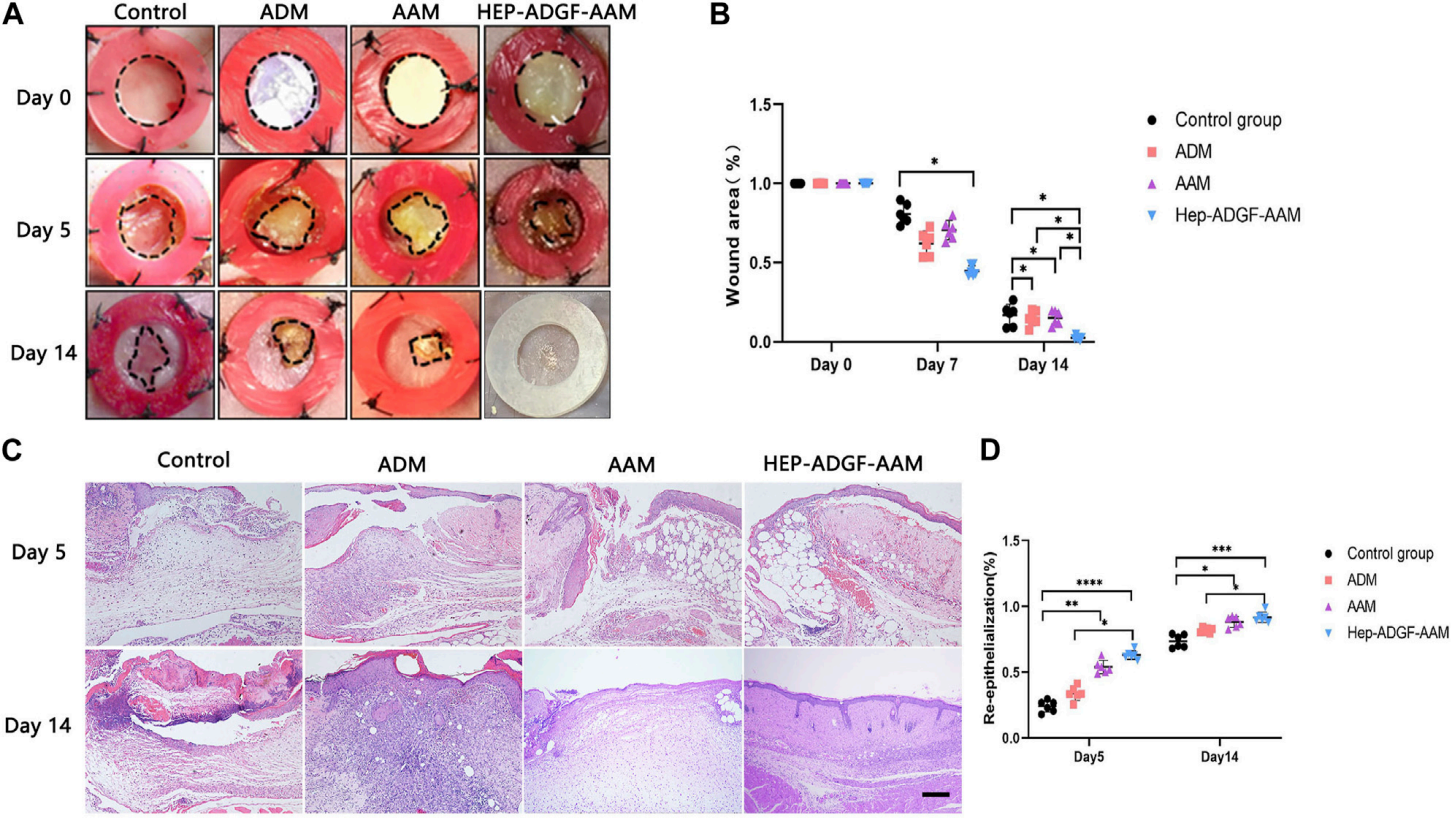
Wound healing efficacies of different acellular biomaterials **(A)** Wounds treated with AAM, ADM, and HEP-ADGF-AAM at different time points. **(B)** Wound healing rate in different treatment groups (*p* < 0.05). **(C)** H&E staining of wounds in different treatment groups. Scale bar = 500 µm. **(D)** Re-epithelialization level of the wounds in different groups, *p < 0.05, **p < 0.01, ***p < 0.001.

Acellular wound dressing was applied to a full-layer wound model of mice. Wound healing was monitored on day 5 and day 14, respectively, and measured by the percentage of lesion area closed. Figure 3A shows representative images of full-layer wound beds of mice in different treatment groups. Histological observations (Figure 3A) showed that on day 5, the wounds treated with HEP-ADGF-AAM showed significant re-epithelialization, while virtually no re-epithelialization was observed in the blank control group. On the 14th day, the blank control group still had unhealed wounds. Compared with a blank control group, AAM, ADM, and HEP-ADGF-AAM all promoted wound healing to varying degrees (Figure 3B, *p* < 0.05). However, there was no significant difference in wound area between the AAM group and the ADM group. Surprisingly, the HEP-ADGF-AAM treatment group achieved almost complete wound healing on day 14, showing the best pro-healing properties. In addition, the level of re-epithelialization induced by HEP-ADGF-AAM at 14 days after injury was significantly higher than that in the control and AAM groups (Figure 3D). HE staining showed (Figure 3C) that granulation tissue, adipose cells, and regenerated skin attachment levels were higher in the HEP-ADGF-AAM group than in the other three groups at day 5 and 14 post-injury. We can see that on day 5, significant subcutaneous fat regeneration can be observed in both ADM and HEP-ADGF-AAM treatment groups. And, unlike the other groups, wound skin in the HEP-ADGF-AAM treated group had more regenerated hair follicles and sebaceous glands at day 14.

### 3.4 HEP-ADGF-AAM enhances fibroblast migration

**FIGURE 4:**
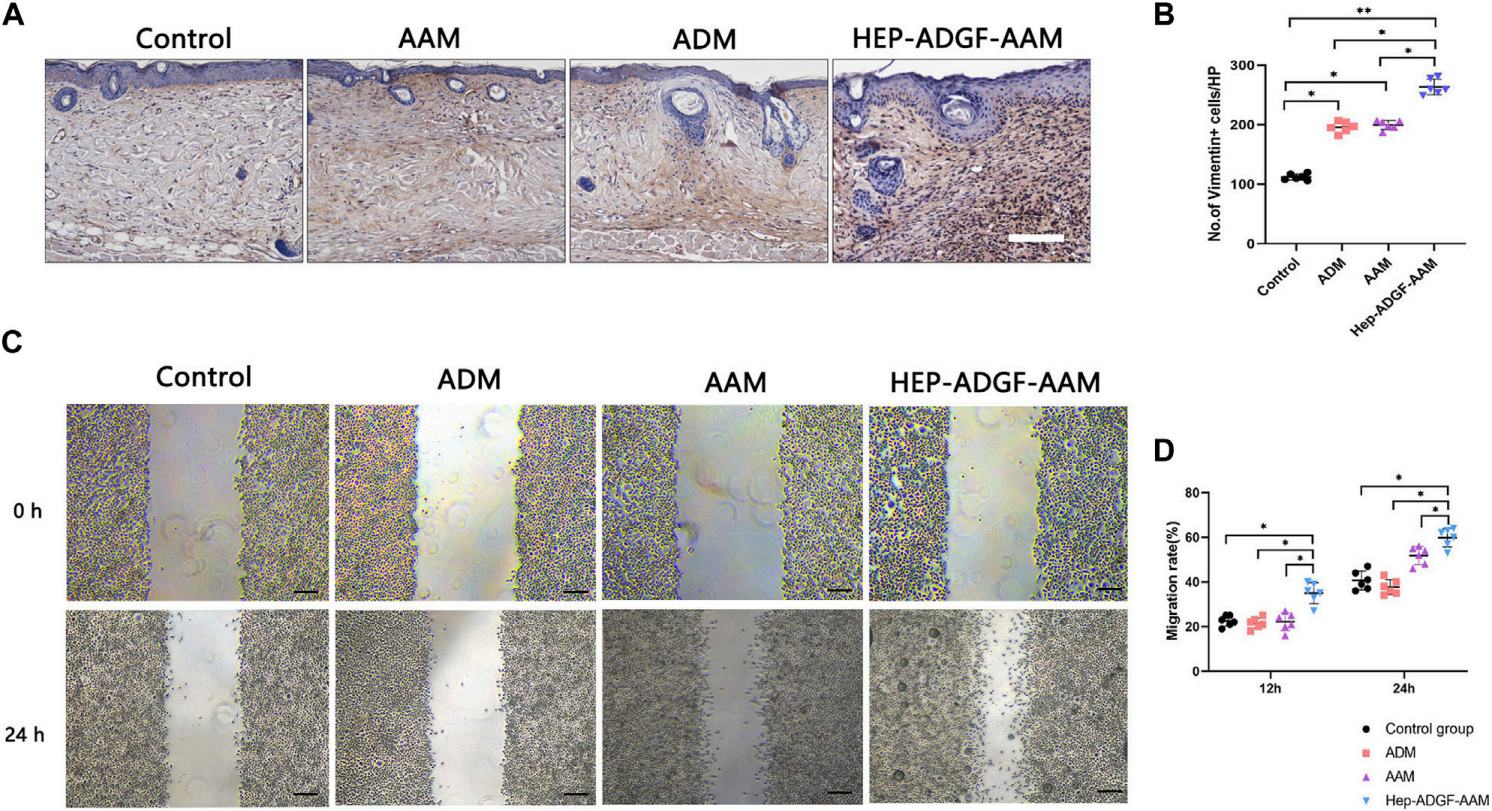
HEP-ADGF-AAM promotes fibroblast migration into the wound bed **(A)** Representative immunohistological staining (vimentin) of the wound bed in the four groups. Scale bar = 500 µm. **(B)** Quantification of vimentin + cells of the wound bed in the four groups. *p < 0.05, **p < 0.01. **(C)** An *in vitro* scratch assay was performed by culturing fibroblasts with soak solution with different acellular products. Scale bar = 100 µm. **(D)** Migration rate of the fibroblasts in the *in vitro* scratch assay. *p < 0.05.

After the skin defect is formed, the repair process begins with the coordination of a variety of cells. The directional migration and proliferation of activated fibroblasts from the dermis to the wound is important for the remodeling of the extracellular matrix in the defective skin. For this purpose, we used Vimentin to track fibroblasts in the dermis. Immunohistochemical staining suggested (Figure 4A) that AAM, ADM, and HEP-ADGF-AAM could effectively promote the migration of fibroblasts to the wound bed on the 5th day after injury. Among them, the number of vimentin-positive fibroblasts in the wound bed of the HEP-ADGF-AAM group was significantly higher than that in AAM, ADM, and control groups (*p* < 0.05) (Figure 4B). In addition, fibroblasts cultured in immersion solution with different acellular products were used for *in vitro* scratch experiments (Figure 4C). The results showed that after 12 and 24 h, the fibroblast migration rate of the HEP-ADGF-AAM group was significantly faster than that of the other three groups. However, fibroblast migration did not differ significantly between ADM, AAM, and negative controls (Figure 4D).

### 3.5 HEP-ADGF-AAM promotes angiogenesis

**FIGURE 5:**
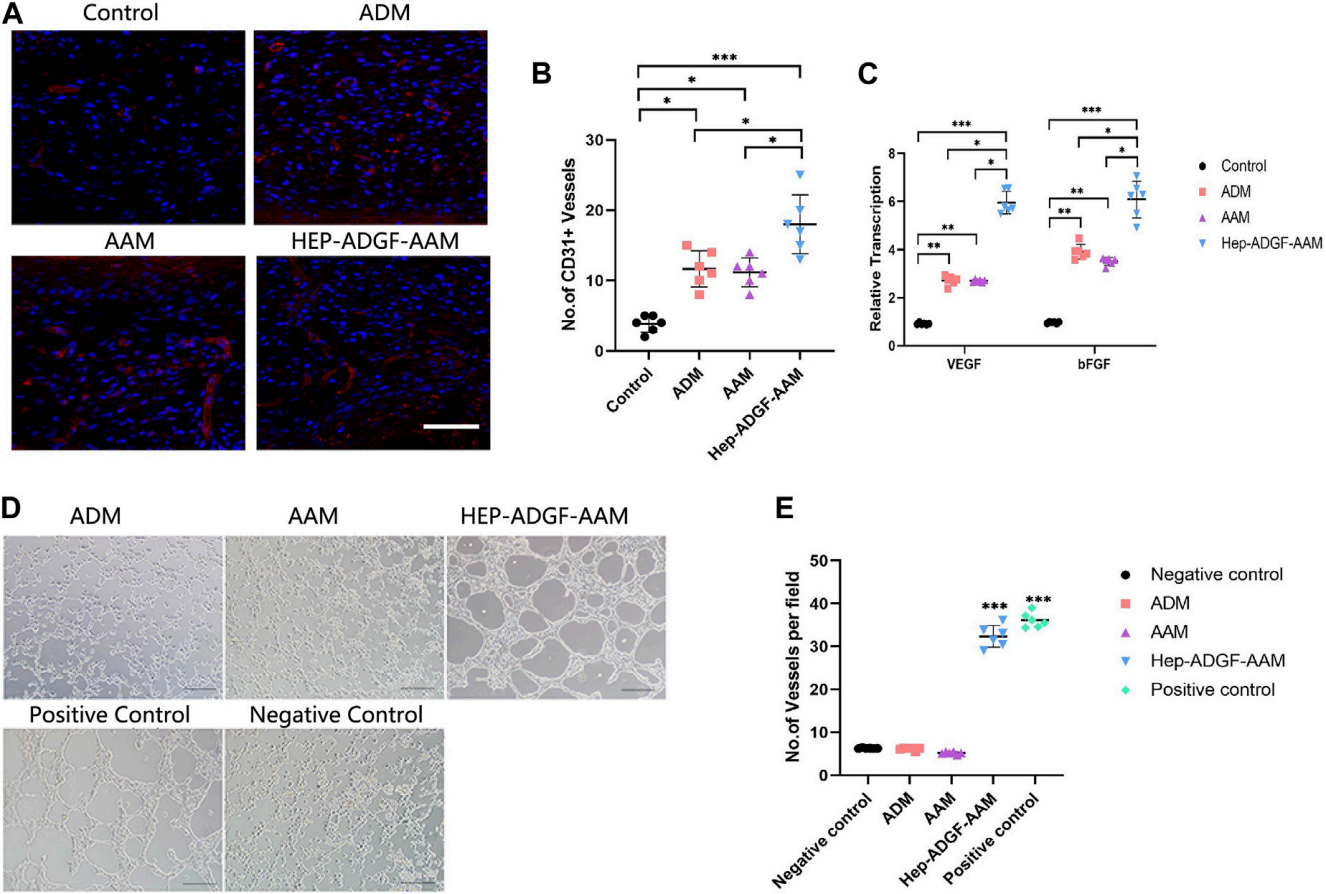
Effects of different acellular products on angiogenesis in the wound bed and tube formation *in vitro*. **(A)** Representative immunofluorescence staining(CD31) of the wound bed in the four groups. Scale bar = 100 µm. **(B)** Quantification of CD31^+^ cells of the wound bed in the four groups. **(C)** Real-time PCR measurements of angiogenic genes (VEGF and bFGF) in the four groups. **(D)** Tubule formation assay by HUVECs with different treatment. Scale bar = 100 µm **(E)** The number of tubular structures in the tubule formation assay. *p < 0.05, **p < 0.01,***p < 0.001.

To explore whether treatment with HEP-ADGF-AAM has the effect of stimulating angiogenesis, we used CD31 immunofluorescence staining to characterize and quantify the number of CD31-positive capillary cells in wounds of different treatment groups. Only a small amount of neovascularization was observed in the blank control group, and treatment with AAM, ADM, and HEP-ADGF-AAM significantly promoted wound angiogenesis (Figure 5A). Quantitative angiogenesis showed that the amount of CD31 in AAM group, ADM group, and HEP-ADGF-AAM group was significantly higher than that in the control group (*p* < 0.05) (Figure 5B). To further confirm their ability to promote vascular regeneration, mRNA levels of angiogenic genes (VEGF and bFGF) in the wound bed were detected by qRT-PCR. The results showed that these levels in the HEP-ADGF-AAM, AAM, and ADM groups were significantly higher than those in the control group (*p* < 0.05) (Figure 5C). In addition, we used *in vitro* tube formation experiments to directly observe the angiogenesis induction ability of HEP-ADGF-AAM on human umbilical vein endothelial cells (HUVECs). In the presence of HEP-ADGF-AAM, the typical tubular structure could be observed after 18h, which was similar to the positive control group and significantly higher than the negative group. However, almost no typical tubular structure was observed when HUVECs were cultured with AAM or ADM throughout the assay process (Figure 5D). Quantitative analysis of capillary tubular structures showed (Figure 5E) that the number of tubular structures in the HUVECsHEP-ADGF-AAM group and positive control group cultured with HEP-ADGF-AAM was significantly higher than that in other groups (*p* < 0.05).

### 3.6 HEP-ADGF-AAM induces adipogenesis and promotes adipogenic differentiation of adipose-derived stem cells (ADSCs) in a paracrine manner

**FIGURE 6:**
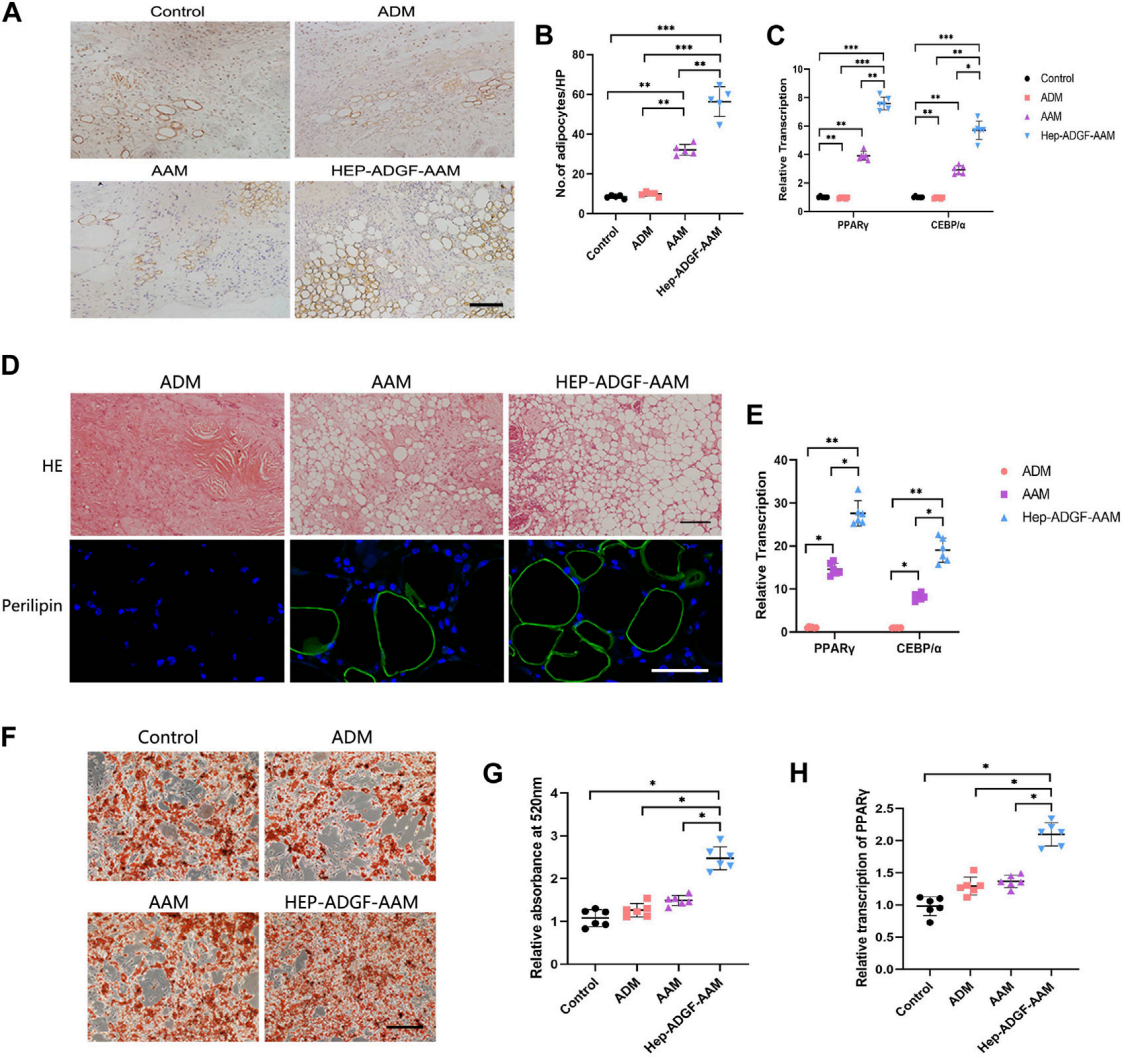
HEP-ADGF-AAM induces adipogenesis and promotes adipogenic differentiation of ADSCs **(A)** Representative immunohistological staining (perilipin) of the wound bed in the four groups. Scale bar = 100 µm. **(B)** TQuantification of perilipin + cells of the wound bed in the four groups. **(C)** Real-time PCR measurements of adipogenic genes (PPARγ and CEBP/α) in the four groups. **(D)** H&E staining and immunofluorescence staining (perilipin) of transplanted tissue in different treatment groups. Scale bar = 50 µm. **(E)** Real-time PCR measurements of adipogenic genes (PPARγ and CEBP/α) in the transplanted tissue. **(F)** Oil Red O staining of ADSCs cultured with acellular products under adipogenic induction conditions. Scale bar = 100 µm. **(G)** Measurement of absorbance at 530 nm of ADSCs cultured with acellular products under adipogenic induction conditions. **(H)** Real-time PCR measurements of adipogenic gene (PPARγ) of ADSCs cultured with acellular products under adipogenic induction conditions. Results with *p < 0.05, **p < 0.01,***p < 0.001 were considered statistically significant.

At Day 5, perilipin + adipocytes were found in the wound bed in all four groups (Figure 6A). The number of perilipin + adipocytes in the wound bed was significantly higher in the HEP-ADGF-AAM group than in the other three groups (*p* < 0.05) (Figure 6B). Expression of adipogenic genes was tested by qRT-PCR. Expression of PPARγ and CEBP/α in wounds was significantly higher in the HEP-ADGF-AAM group than in the other three groups (*p* < 0.05) (Figure 6C). To evaluate the adipogenic capacities of the acellular products, they were subcutaneously transplanted into nude mice. After 12 weeks, H&E staining showed that grafts in the ADM group did not contain adipocytes, and this was confirmed by immunofluorescence staining of perilipin. Viable adipocytes (perilipin+) were found in the AAM and HEP-ADGF-AAM groups (Figure 6D). mRNA levels of adipogenic genes (PPARγ and CEBP/α) in the grafts were tested by qRT-PCR. Adipogenic gene expression was significantly higher in the HEP-ADGF-AAM and AAM groups than in the ADM group (*p* < 0.05) (Figure 6E). Oil Red O staining was performed following culture of ADSCs with the acellular products under adipogenic induction conditions (Figure 6F). Measurement of absorbance at 530 nm showed that HEP-ADGF-AAM improved adipogenic differentiation, while AAM and ADM did not (Figure 6G). In addition, the mRNA level of PPARγ was significantly higher in the HEP-ADGF-AAM group than in the other three groups (*p* < 0.05) (Figure 6H).

## 4 Discussion

**FIGURE 7:**
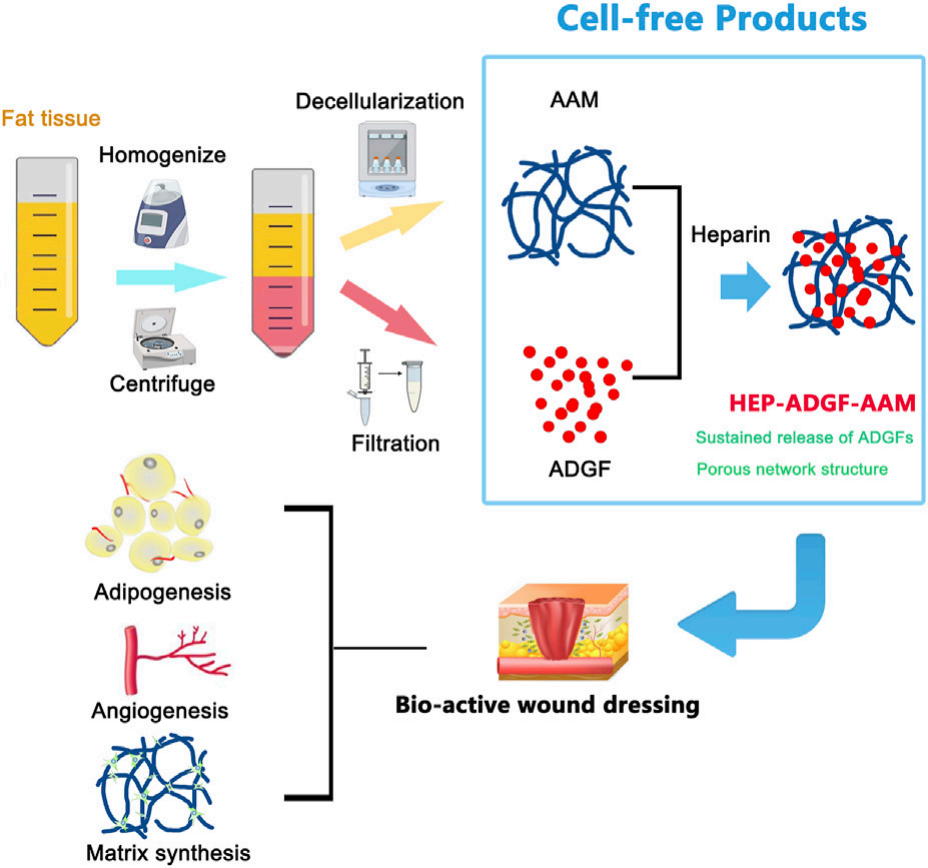
Graphical conclusion of the construction and application of HEP-ADGF-AAM. Both AAM and ADGFs were obtained from human adipose tissue via different procedures. The obtained adipose tissue was first processed through homogenization and centrifugation. The adipose layer underwent repeated freeze-thawing and high-speed physical homogenization to obtain AAM. The liquid layer underwent filtration to obtain ADGFs. These two adipose tissue-derived products were then assembled into a biofunctional wound dressing with the assistance of heparin. The HEP-ADGF-AAM is a cell-free product that could be a promising therapeutic biomaterial for skin wound healing. (AAM, adipose acellular matrix; ADGFs, adipose-derived growth factors; HEP-ADGF-AAM, heparinized ADGF-bound AAM).

Large and deep wounds including surgical wounds and burns of both partial and full thickness require bio-functional dressings or dermal substitutes to replace lost tissue and support intrinsic self-regeneration mechanisms (Zhong et al., 2010; Goodarzi et al., 2018). New developments in ECM-derived tissue-engineered scaffolds may achieve the ultimate goal of tissue regeneration rather than tissue replacement (Yi et al., 2017; Varshosaz et al., 2019). Considering that recent studies have provided evidence that adipocytes and an adipogenic microenvironment provide essential support for skin repair and regeneration (Schmidt and Horsley, 2013; Franz et al., 2018; Shook et al., 2020), we fabricated a biomaterial called HEP-ADGF-AAM from adipose tissue to support wound healing. In this study, we obtained AAM and ADGFs from human adipose tissue. These two products were then assembled into a bio-functional wound dressing using heparin. The final product, HEP-ADGF-AAM, contained adipose ECM and stably released ADGFs. In a full-thickness wound mouse model, HEP-ADGF-AAM significantly improved wound closure and angiogenesis. Furthermore, HEP-ADGF-AAM potently induced adipogenesis in the wound bed (Figure 7).

The manufacturing process of AAM varies widely due to the lack of a standardized preparation scheme. Several studies have reported alternative methods to efficiently decellularize adipose tissue (Brown et al., 2011; Choi et al., 2011; Song et al., 2018). Methods for tissue decellularization differ depending on the intended application of AAM. In the present study, AAM was prepared by repeated freeze–thaw cycles followed by high-speed physical homogenization. Mechanical homogenization is a rapid and efficient method to remove lipids from adipose tissue, reduces the use of harsh chemical agents, and may improve the retention of ECM components.

Decellularization of adipose tissue reduces the diversity and levels of proteins in AAM. Surface modification with heparin improves the capacity of collagen scaffolds to bind growth factors (Wissink et al., 2000b; Wissink et al., 2001; Pieper et al., 2002). Our previous study showed that ADGFs effectively promote wound healing by enhancing fibroblast migration, vessel formation, and intradermal adipogenesis (He et al., 2019). In this study, we used heparinized AAM to physically immobilize ADGFs, which were removed from AAM during the decellularization process. ADGFs were released from HEP-ADGF-AAM in a controlled fashion. Our results confirmed that relatively high local concentrations of VEGF, bFGF, and HGF released from HEP-ADGF-AAM, but not ADGF-AAM, were stably maintained. The *in vitro* scratch assay and angiogenesis induction test showed that HEP-ADGF-AAM stimulated fibroblast migration and tube formation by endothelial cells in a paracrine manner, while ADM and AAM did not. These results suggest that HEP-ADGF-AAM stably releases ADGFs and improves cellular functions in a paracrine manner.

Heparin was reported to improve the porous structure and maintain good mechanical properties of the scaffolds. Furthermore, the combination of heparin and growth factors into the scaffold can significantly improve the microenvironments of cell growth and increase the secretion function of cells (Fan and Yang, 2017). It is also reported that the use of heparin can increase the biocompatibility of implanted materials and growth factors with a high affinity, which will protect the protein from degradation or denaturation and augmented the biological activity of growth factors (Park et al., 2018). What’s more, cells on the heparin-conjugated scaffold exhibit high bioconpatibility and could maintain an effective local concentration of growth factors on the scaffold surface, which also verify the effectiveness of heparin and hopeful prospects (Ikegami and Ijima, 2020).

Adipocytes are traditionally considered to be important energy storage cells and were recently found to have essential functions for skin homeostasis and wound healing (Schmidt and Horsley, 2013; Wang et al., 2018; Franz et al., 2018; Shook et al., 2020). Migration and repopulation of adipocytes may be essential for wound healing. Adipocyte precursor cells proliferate, and mature adipocytes repopulate skin wounds following inflammation and in parallel with fibroblast migration (Schmidt and Horsley, 2013; Franz et al., 2018; Guerrero-Juarez et al., 2019; Shook et al., 2020). In this study, mature adipocytes were observed in the wound bed in the control group, indicative of adipocyte recruitment or spontaneous regeneration during wound healing. Moreover, AAM and HEP-ADGF-AAM increased the number of adipocytes in the wound bed. We compared the adipogenic capacities of these acellular products after transplantation. ADM did not induce adipocyte regeneration *in situ*, while AAM and HEP-ADGF-AAM stimulated adipogenesis, and HEP-ADGF-AAM had the best adipogenic capacity after transplantation. The interaction between biological dressings and wounds may be dependent on direct contact or paracrine mechanisms. ADSCs were cultured with these acellular products during adipogenic induction to verify the effects of growth factors released by these products. The results suggested that only HEP-ADGF-AAM was able to enhance adipogenesis of ADSCs, suggesting that only HEP-ADGF-AAM can release ADGFs to create an adipogenic niche. In summary, ADM is unable to induce adipocyte regeneration, while AAM provides an adipogenic scaffold that induces adipogenesis, and HEP-ADGF-AAM not only functions as an adipogenic scaffold, but also provides an adipogenic niche in a paracrine manner.

The study has some limitations to consider. First, HEP-ADGF-AAM is derived from human adipose tissue, so the differences in tissue activity between different donors and the potential risk of infectious disease transmission cannot be ignored. However, this problem can be solved by rigorous screening for infectious diseases and careful evaluation and selection of donors. Secondly, in this experiment, we used a mouse wound model to demonstrate the excellent efficacy of the lipogenic biomaterials HEP-ADGF-AAM in promoting wound healing. However, major structural and functional differences between mouse skin and human epidermis limit the inference of the results of this therapeutic model. To this end, we tested human cells directly instead of animal cells *in vitro* to minimize this limitation. However, their wound-healing mechanisms are different. At present, the biggest limitation of the mouse wound healing model is that the wound closure in mice is mainly through contraction, while the wound closure in normal people is mainly through re-epithelialization. To this end, attempts can be made to avoid contraction-mediated wound healing by removing the thin striated muscle located between the subcutaneous fat and the dermis during wound modeling, thus mimicking the wound healing process in human skin. In addition, the development of reliable humanized skin wound models in recent years has also provided a more reliable platform for testing skin repair strategies. All in all, more robust preclinical studies are still needed to further confirm the benefits observed in mouse models. In addition, nude mice were used as experimental objects in this study, which could not effectively evaluate the effect of allograft products due to immune deficiency.

## 5 Conclusion

In this study, we showed that HEP-ADGF-AAM accelerates wound epithelialization and angiogenesis, and provides an adipogenic microenvironment for fibroblast activation and vessel formation. This study demonstrated that HEP-ADGF-AAM has a better therapeutic effect than ADM, which has been clinically used for many years. Overall, the results of this study indicate that the adipogenic biomaterial HEP-ADGF-AAM accelerates wound healing by inducing adipocyte regeneration and enhancing angiogenesis and re-epithelialization. Further studies are needed to confirm the role of the adipogenic microenvironment in skin wound healing.

## Data Availability

The original contributions presented in the study are included in the article/Supplementary Material, further inquiries can be directed to the corresponding authors.
